# Extraordinary Case of Pericarditis

**Published:** 1843-05

**Authors:** Chester G. Ballard

**Affiliations:** Greencastle, Indiana


ART. II.—*Extraordinary case of Pericarditis*. By CHESTER G. BALLARD, M. D., of Greencastle, Indiana.

I have denominated the following case extraordinary from the fact that I have neither met with the like, nor in my intercourse with the profession have I had one of the kind reported to me—and after the most diligent search through many volumes of Medical Journals, and all the standard works in my possession, I am unable to find an analagous case.

About three weeks since I was called in the night to visit an orphan male child between five and six years old, at the residence of its grand-mother in this place, in consultation with the attending physican, who informed me that the child had been under his treatment for several days with the following symptoms: slight fever, coated tongue, tumid abdomen, slight cough, restlessness, and constant picking of the nose. Treatment—vermifuge medicines, calomel and Dover's powder. Under this medication the febrile symptoms had been to some extent relieved, but no worms dislodged.

The cause of alarm at this hour, was sudden dyspnœa and restlessness, which however had much abated before my arrival. I found the mucous membrane of the nose, mouth, and fauces in a high state of irritation—pulse feeble and quick, but not intermitting—abdomen very tense, without the least apparent tenderness—fluctuation distinct. I learned from the family that the child had been fretful, peevish, and sickly, since it was seven or eight months old, about which time it lost its mother. I also understood that it had been subject to severe spells of crying, at the same time manifesting the strongest indications of pain, which, when its judgment was sufficiently matured, it always located by putting its hand upon the sternum. Walking up hill, or the playful exercises of childhood always produced difficulty of breathing—appetite had been uniformly good, and bowels regular; but notwithstanding its uniform appetite and the healthy function of all the chylopoietic viscera, marasmus had set its signet upon him, and marked him for a victim.

I gave it as my decided conviction there was serous effusion in both the great cavities, and that worms had nothing to do in the matter—and that death was inevitable. I advised the gentleman in attendance to apply an epispastic from the upper part of the sternum to the umbilicus—to give small portions of jalap and cream of tartar at proper intervals until catharsis was established, and support the strength of his patient with appropriate means if occasion should require.

Following day—blister looked fine—several dark, bilious dejections from the bowels—breathing short but easy as common—mucous irritation almost disappeared—copious secretion from the kidneys, which were torpid the night before—abdominal tension much less. After which I saw him no more until I was requested by the physician to make a post-mortem examination, which was done sixteen hours after death.

*Autopsy.*—When separating the cartilages of the ribs in removing the sternum, there was a discharge of about half a pint of pale yellow serum containing no flocculi—this I was prepared to see—sternum being removed, the pericardium presented itself to view, filling almost the entire cavity of the thorax, which I supposed to contain a similar fluid, but on making an incision I was surprised to find it filled with healthy looking pus, and to the enormous amount at least of two quarts. The lamina of the sac were greatly thickened, the outer one seemed completely united with the mediastinum—its attachment to the cordiform tendon of the diaphragm much extended—the lining membrane rather more pallid than natural—no lesion in its structure. The heart was natural in color, its ventricles and valves all sound, but it was soft; the softening however appeared to be nothing more than a tendency to general decay—all the muscles were flaccid. Lungs collapsed and contracted until neither lobe would measure more than two and a half inches from base to apex, and in external appearance resembling glandular bodies, and possessing, when submitted to the test of the senses, about the same density as that of the spleen—they exhibited no marks of past or present inflammation, and could not, I should presume, by the greatest expansion of which they were capable, have contained more than three or at most four cubic inches of atmospheric air—the pleura on both sides possessed all the firmness and brilliancy of that of the most healthy subject. The cause of the serum in the chest was probably deficiency of absorption—as also that of the abdomen, which also contained upwards of a pint of colourless serum. The abdominal viscera were all sound.

The great singularity of this case seems to consist in three particulars, viz: the length of time the disease had existed; the serous membrane of the pericardium putting on suppurative action so perfectly; and the enormous quantity of pus secreted. That it was insidious in its character should not be thought an anomaly, for that is one of its striking characteristics. Laennec acknowledges that it is as frequently mistaken as recognised, even by himself. With regard to the slow progress of the disease in the above case, we think there is no reasonable doubt, when we consider the sickly character of the child from the time it was seven or eight months old up to the day of its death; constantly complaining of pain in the thorax, and shortness of breath following the least active exertion. An argument might be drawn with great force to sustain this position from the condition of the lungs. But the serous membrane producing such perfect purulent secretion and to such extent, is what we think the most singular feature in the case. Dewees remarks that the pericardium acts from laws of its own; this case seems to favor that opinion. The cough mentioned at our first visit, was by no means a concomitant of the chronic disease. The only acute disease with which it was ever afflicted, that of mucous irritation, was always accompanied with a slight cough.

December, 1842.

